# A comparative study of excitation/inhibition balance analysis methods and their connection to Alzheimer's disease development

**DOI:** 10.1002/alz70856_102214

**Published:** 2025-12-24

**Authors:** Adrián Quivira‐Lopesino, Pablo Cuesta, Isabel Suárez, Fernando Maestú

**Affiliations:** ^1^ Universidad Complutense de Madrid, Madrid, Madrid, Spain

## Abstract

**Background:**

Alzheimer's Disease (AD) is the most common form of dementia worldwide. Its neuropathology is characterized by the accumulation of Aβ peptides and hyperphosphorylated tau, leading to alterations in the brain's excitatory and inhibitory connections. This results in a disruption of the excitation/inhibition (E/I) balance, which plays a key role in information encoding, synaptic plasticity, and memory stability.

**Method:**

Recently, researchers at the University of California San Diego (UCSD) developed an algorithm called FOOOF (Fitting Oscillations & One Over F), capable of determining the aperiodic exponent (ExpA) of the power spectrum, which has been linked to the E/I balance. Meanwhile, researchers at the University of Amsterdam (UvA) have developed a method to quantify the functional E/I ratio (fE/I) of neural oscillations. Thus, analyzing the E/I balance using these techniques, applied to MEG signals from individuals with mild cognitive impairment (MCI) and healthy participants (HP), could provide valuable insights into how this disease progresses at the electrophysiological level.

**Result:**

In the early stages of MCI, the accumulation of neuritic plaques promotes the loss of GABAergic neurons, leading to hyperexcitation (higher E/I balance). However, as the disease progresses and neurofibrillary tangles begin to accumulate, neuronal activity decreases (lower E/I balance). Analyses with the first methodology revealed that MCI patients exhibited overall higher ExpA, suggesting a lower E/I balance. Analyses with the second methodology showed that MCI patients had fE/I ratio values below 1, indicating a shift in the E/I balance toward inhibition.

**Conclusion:**

The results obtained in this study demonstrate that both methods effectively measure approximations of the E/I balance. Furthermore, both methodologies seem to indicate a decrease in the E/I balance in individuals with MCI, which could be associated with the characteristic accumulation of hyperphosphorylated tau protein observed in AD. The implications of these results are still under investigation, but these findings could be useful to understand the progression of AD.

